# Galectin-1 Overexpression Activates the FAK/PI3K/AKT/mTOR Pathway and Is Correlated with Upper Urinary Urothelial Carcinoma Progression and Survival

**DOI:** 10.3390/cells9040806

**Published:** 2020-03-26

**Authors:** Yu-Li Su, Hao-Lun Luo, Chun-Chieh Huang, Ting-Ting Liu, Eng-Yen Huang, Ming-Tse Sung, Jen-Jie Lin, Po-Hui Chiang, Yen-Ta Chen, Chih-Hsiung Kang, Yuan-Tso Cheng

**Affiliations:** 1 Division of Hematology Oncology, Department of Internal Medicine, Kaohsiung Chang Gung Memorial Hospital and Chang Gung University, College of Medicine, Kaohsiung 833, Taiwan; 2 Clinical Trial Center, Kaohsiung Chang Gung Memorial Hospital, Kaohsiung 833, Taiwan; 3 Department of Urology, Kaohsiung Chang Gung Memorial Hospital and Chang Gung University, College of Medicine, Kaohsiung 833, Taiwan; 4 Department of Radiation Oncology, Kaohsiung Chang Gung Memorial Hospital and Chang Gung University, College of Medicine, Kaohsiung 833, Taiwan; 5 Department of Pathology, Kaohsiung Chang Gung Memorial Hospital and Chang Gung University, College of Medicine, Kaohsiung 833, Taiwan

**Keywords:** galectin-1, upper urinary urothelial carcinoma, epithelial–mesenchymal transition, survival, phosphoinositide 3-kinases, focal adhesion kinase, mammalian target of rapamycin

## Abstract

Galectin-1 (GAL1) is a β-galactoside-binding protein involved in multiple aspects of tumorigenesis. However, the biological role of GAL1 in upper tract urothelial carcinoma (UTUC) has not been entirely understood. Herein, we investigated the oncological effects of GAL1 expression in tumor specimens and identified related gene alterations through molecular analysis of GAL1. Clinical parameter data and tumor specimens were collected from 86 patients with pT3N0M0 UTUC who had undergone radical nephroureterectomy. We analyzed the difference in survival by using Kaplan–Meier analyses and Cox proportional regression models and in GAL1 expression by using immunohistochemical (IHC) methods. Public genomic data from the Cancer Genome Atlas (TCGA) and GSE32894 data sets were analyzed for comparison. Using four urothelial carcinoma (UC) cell lines (BFTC-909, T24, RT4, and J82) as in vitro models, we evaluated the functions of GAL1 in UC cell growth, invasiveness, and migration and its role in downstream signaling pathways. The study population was classified into two groups, GAL1-high (n = 35) and GAL1-low (GAL1 n = 51), according to IHC interpretation. Univariate analysis revealed that high GAL1 expression was significantly associated with poor recurrence-free survival (RFS; *p* = 0.028) and low cancer-specific survival (CSS; *p* = 0.025). Multivariate analysis revealed that GAL1-high was an independent predictive factor for RFS (hazard ratio (HR) 2.43; 95% confidence interval (CI) 1.17–5.05, *p* = 0.018) and CSS (HR 4.04; 95% CI 1.25–13.03, *p* = 0.019). In vitro studies revealed that GAL1 knockdown significantly reduced migration and invasiveness in UTUC (BFTC-909) and bladder cancer cells (T24). GAL1 knockdown significantly reduced protein levels of matrix metalloproteinase-2 (MMP-2) and MMP-9, which increased tissue inhibitor of metalloproteinase-1 (TIMP-1) and promoted epithelial–mesenchymal transition (EMT). Through gene expression microarray analysis of GAL1 vector and GAL1-KD cells, we identified multiple significant signaling pathways including p53, Forkhead box O (FOXO), and phosphoinositide 3-kinase/protein kinase B (PI3K/AKT). We validated microarray results through immunoblotting, thus proving that downregulation of GAL1 reduced focal adhesion kinase (FAK), p-PI3K, p-AKT, and p-mTOR expression. We concluded that GAL1 expression was highly related to oncological survival in patients with locally advanced UTUC. GAL1 promoted UC invasion and metastasis by activating the FAK/PI3K/AKT/mTOR pathway.

## 1. Introduction

Upper tract urothelial carcinoma (UTUC) is an aggressive and lethal disease. The incidence of UTUC in Western countries is relatively low (5% of all urothelial carcinoma); however, the prognosis of UTUC is considerably worse than that at the same pathological stage of urothelial carcinoma of the bladder (UCB) [1,2,3]. The incidence of UTUC in Taiwan is considerably higher than the worldwide incidence (30%–40% of all urothelial carcinoma cells (UCs)), which indicates that some unknown carcinogenic or environmental factors contribute to tumor development and growth [4,5,6,7]. Histologically, UCs arising from the upper urinary tract and urinary bladder are grossly identical. However, several comprehensive genomic studies have argued that UTUC and UCB are distinct and that the disease can be characterized by a unique fingerprint mutation signature; this signature takes the form of A:T to T:A transversions induced by aristolochic acid [7]. By contrast, specific insights have been gained by studying an autosomal dominant familial syndrome, namely Lynch syndrome (hereditary nonpolyposis colorectal cancer), which is associated with an increased risk of UTUC [8]. Overall, the carcinogenesis and molecular biology of UTUC and UCB may not involve identical pathways, and both cancers should be treated and discussed separately.

Treatment of patients with UTUC often requires multidisciplinary teams, consisting of urologists, medical oncologists, and radiation oncologists. Radical nephroureterectomy with excision of an ipsilateral bladder cuff is the gold standard treatment for organ-confined UTUC [9]. Early stages of UTUC can be cured by radical surgery; however, disease recurrence and distant metastasis occur commonly in the advanced stages of UTUC (T3 or T4), which is incurable and inevitably results in patient death. Currently, studies with consistent results of adjuvant treatment of locally advanced UTUC and randomized trials to guide postoperative management are not available. Moreover, the comprehensive pathogenesis and molecular features of UTUC are currently under investigation [10]; hence, developing effective treatments is difficult.

Galectin-1 (GAL1) is a β-galactoside-binding protein encoded by *LGALS1* on chromosome 22q12 and participates in multiple aspects of tumorigenesis, including cell proliferation, invasiveness, metastasis, and angiogenesis [11,12,13,14,15]. GAL1 expression has been frequently reported to increase in several types of tumors, including those of the colon, breast, lung, and uterine cervix [16,17,18,19] as well as those in Hodgkin lymphoma [20]. Moreover, higher expressions of GAL1 in gastric and cervical cancer have been reported to be positively correlated with advanced tumor stage, tumor invasion, and lymph node metastasis [21,22]. In terms of prognostic effect, several anecdotal studies have demonstrated a consistent relationship between high GAL1 expression and poor survival in patients with cancers of the lung, uterine cervix, and bladder [23,24,25]. Shen et al. demonstrated that the interplay between GAL1 and bladder cancer invasiveness and that between GAL1 and progression was mediated via the Ras-Rac1-MEKK4-JNK-AP1 signaling pathway [26]. In the lung cancer model, downregulation of GAL1 reduced tumor invasion and migration via the p38 MAPK-ERK and cyclooxygenase-2 (COX2) pathways [27]. Although some previous studies have confirmed the crucial role of GAL1 in tumorigenesis and drug resistance pathways, the role of GAL1 in UTUC remains unknown and has not been investigated thus far.

In the present study, we examined the prognostic role of GAL1 in patients with locally advanced UTUC (pT3). Furthermore, we evaluated the biological roles of GAL1 in UTUC and UCB cell lines and attempted to decipher the GAL1 mediating downstream oncological pathways in UTUC.

## 2. Materials and Methods 

### 2.1. Antibodies and Reagents

Many reagents, including Dulbecco’s modified Eagle’s medium (DMEM), McCoy’s 5a medium, trypsin-ethylenediaminetetraacetic acid, fetal bovine serum (FBS), and phosphate-buffered saline (PBS), were obtained from Biowest (Nuaillé, France). Polyvinylidene difluoride (PVDF) membranes, and goat anti-rabbit and horseradish peroxidase (HRP)-conjugated immunoglobulin (Ig) G were obtained from Millipore (Billerica, MA, USA). Protease inhibitor cocktail and DMSO were obtained from BioSource International (Camarillo, CA, USA). Cell extraction radioimmunoprecipitation assay (RIPA) buffer was obtained from TOOLS (TOOLS, Taiwan). Enhanced chemiluminescence (ECL) Western blotting reagents were obtained from Pierce Biotechnology (Rockford, IL, USA). Mouse anti-human β-actin antibodies were obtained from Sigma (St Louis, MO, USA). Rabbit anti-human FAK, mTOR, and p-mTOR antibodies were obtained from Epitomics (Burlingame, CA, USA). Rabbit anti-human TIMP-1, AKT, and p-AKT antibodies were obtained from ProteinTech Group (Chicago, IL, USA). Rabbit anti-human MMP-2, MMP-9, PI3K, p-PI3K, and EMT kit (#9782) antibodies were obtained from Cell Signaling Technology (Danvers, MA, USA).

### 2.2. Patients and Tumor Samples

We enrolled 86 patients with UTUC who had undergone radical nephroureterectomy and bladder cuff excision with final pathologically confirmed as pT3N0 stage between January 2005 and December 2012 in Kaohsiung Chang Gung Memorial Hospital (KSCGMH). Preoperative and pathological features of eligible patients, including age, sex, comorbidity, pathological TNM stage, histopathological subtypes and variants, presence of lymphovascular invasion (LVI) or perineural invasion, pattern of tumor formation (papillary or infiltrative), solitary or multicentric, presence of hydronephrosis, type of disease recurrence (local, regional, distant, or urinary tract recurrence), and date of disease recurrence and death, were recorded in detail. All clinicopathological data were collected retrospectively by accessing an electronic medical record system. All surgical tumor samples were fixed in 10% formalin and embedded in paraffin. All study procedures performed in this research were approved by the Chang Gung Medical Foundation Institutional Review Board (No. 104-5487B). Owing to retrospective nature, human tumor specimens of upper tract urothelial carcinoma were obtained from patients undergoing radical nephroureterectomy at Kaohsiung Chang Gung Memorial Hospital.

### 2.3. IHC Analysis

Immunohistochemical (IHC) staining for detecting GAL1 was performed in all resected tumor specimens. The paraffin-embedded tumor tissues were cut to obtain 4 mm thick sections. Briefly, after deparaffinization and rehydration, the sections were subjected to heat-induced epitope retrieval in 10 mM citrate buffer (pH 6.0) in a hot water bath (95 °C) for 20 min. After blocking with 1% goat serum for 1 h at room temperature, the sections were incubated with primary antibodies for at least 18 h at 4 °C. GAL1 protein expression was detected using a primary antibody specific to Gal1 (H-45, Santa Cruz Biotechnology, Santa Cruz, CA, USA); subsequently, the sections were incubated with the secondary antibody (Histofine MAX PO, Nichirei, Tokyo, Japan) for 30 min. Immunodetection was performed using the LSAB2 kit (Dako, Carpinteria, CA) followed by 3-3′-diaminobenzidine for color development and hematoxylin for counterstaining. An incubation mixture in which the primary antibody was replaced by PBS was used as a negative control. All sections were scored for GAL1 cytoplasmic expression, and the results were interpreted by two independent pathologists (M.T.S. and T.T.L.) after blinding. We determined 10% expression as the optimal cutoff level. Tumors exhibiting <10% expression of GAL1 were classified as low expression.

### 2.4. Cell Lines and Culture

Human UC cell lines, namely the BFTC-909 (renal pelvis), J82 (bladder), RT4 (bladder), and T24 (bladder), were purchased from the Food Industry Research and Development Institute (Hsinchu, Taiwan). The BFTC-909 and J82 cells were cultured in DMEM. The T24 and RT4 cells were cultured in McCoy’s 5a medium, supplemented with 10% FBS and antibiotics (100 U/mL penicillin and 100 μg/mL streptomycin). All cells were incubated in a humidified atmosphere containing 95% air and 5% CO_2_ at 37 °C.

### 2.5. Immunoblotting

For Western blotting, 5 × 10^6^ BFTC-909 cells were seeded in 10-cm plates and were lysed using a cell extraction RIPA buffer. Proteins (25 μg) extracted from the whole cells were separated through 12.5% SDS gel electrophoresis and then transferred onto a PVDF membrane (Millipore) for 2 h at 400 mA using Transphor TE 22 transfer tank (Hoeffer). The PVDF membranes were then incubated with appropriate rabbit polyclonal antibodies at 4 °C for 2 h or overnight. The membranes were washed five times in PBS buffer containing 0.05% Tween 20 and then probed with goat anti-rabbit HRP-conjugated antibody (1:5000) for 1 h. The blots were then visualized using ECL Western Blotting Reagents (Pierce, Rockford, IL, USA).

### 2.6. GAL1 Knockdown Cells with shRNA

For shRNA transfection, 1 × 10^5^ cells were seeded on 3-cm plates and incubated for 24 h at 37 °C. Next, the cells were transfected with GAL1 shRNA or respective controls by using lipofectamine 2000 and incubated for 48 h in a serum-free medium. The cells were transferred to a 10 cm dish for growth, addition of antibiotics, and removal of nontransfected cells.

### 2.7. Stable Knockdown of GAL1 by Using Lentiviruses

We inserted human *LGALS1* (GenBank accession number NM_002305) into the VSV-G pseudotyped lentiviral vectors (Academia Sinica, Taiwan) to silence the expression of GAL1. The two shRNA sequences were as follows:

Gal1sh1 (TRCN0000433733) (5′-CCGGACGGTGACTTCAAGATCAAATCTCGAGATTTGATCTTGAAGTCACCGTTTTTTTG-3′); Gal1sh2 (TRCN0000057425) (5′-CCGGCCTGAATCTCAAACCTGGAGACTCGAGTCTCCAGGTTTGAGATTCAGGTTTTTG-3′); VSV-G, a pseudotyped lentiviral vector, was constructed to silence the expression of GAL1. Furthermore, a negative control vector containing the cytomegalovirus promoter and expressing high levels of green fluorescent protein was also designed. The negative control was also created using a VSV-G. The lentiviral vectors were transfected into the BFTC-909 cells at a multiplicity of infection ranging from 1 to 10 in the presence of 5 μg/mL polybrene (Sigma-Aldrich, St. Louis, MO, USA).

### 2.8. Transwell Migration and Invasion Assay

The BFTC-909 cells and knockdown GAL1 cells were seeded into a transwell insert (Neuro Probe, Cabin John, MD, USA) at 1 × 10^4^ cells/well in serum-free media. The cells were incubated at 37 °C for 24 h to allow cell migration. For invasion assay, 20 μL Matrigel (BD Biosciences, MA, USA) was coated onto polycarbonate membrane filters of 8 μm pore-size, and BFTC-909 and knockdown GAL1 cells were plated in the upper chamber of the Matrigel-coated transwell insert. The migrated and invaded cells on the lower chamber were fixed with 100% methanol and stained with 0.1% crystal violet. Cell numbers were counted using a 100× light microscope.

### 2.9. RNA Isolation and Quantitive PCR

Total RNA was extracted from the cell lines using QIAGEN RNA purification kit. The total RNA (5 μg) was then reverse transcribed using RevertAidTM H Minus Reverse Transcriptase (Fermentas, Waltham, MA, USA). Real-time PCR was performed using SYBR Green PCR master mix (Life Technologies, Carlsbad, CA, USA) and ABI 7500 sequence detection system (Life Technologies). 

Real-time PCR primers used in this study were as follows: 

GAL1 forward: 5′- AGCAGCGGGAGGCTGTCTTTC-3′; 

GAL1 reverse primer: 5′- ATCCATCTGGCAGCTTGACGGT-3′. 

GAPDH forward: 5′-GTCTCCTCTGACTTCAACAGCG-3′; 

GAPDH reverse primer: 5′-ACCACCCTGTTGCTGTAGCCAA-3′. 

All primers were purchased from OriGene (Rockville, MD, USA) and checked for specificity using BLAST (NCBI). Exon and intron junctions were spanned.

### 2.10. Gene Expression Microarray Assay

Total RNA extraction from peripheral blood mononuclear cells (PBMCs) was performed using a miRNeasy mini kit following the manufacturer’s protocol (Qiagen GmbH, Hilden, Germany). Furthermore, cRNA preparation, sample hybridization, and scanning were performed following the protocols provided by Affymetrix (Affymetrix, Santa Clara, CA, USA) and Cogentech Affymetrix microarray unit (Campus IFOM IEO, Milan, Italy). All samples were hybridized on a Human Clariom D (Thermo Fisher Scientific) gene chip and were analyzed using the Transcriptome Analysis Console 4.0 software (Applied Biosystem, Foster City, CA, USA by Thermo Fisher Scientific, Waltham, MA, USA). Human Clariom D arrays enable investigation of more than 540,000 transcripts sourced from the largest public databases starting from as little as 100 pg of total RNA. Relative gene expression levels of each transcript were validated by applying a one-way analysis of variance (*p* ≤ 0.01) and multiple testing corrections. Coding genes and lncRNAs that displayed an expression level at least 1.5-fold different in the test sample versus control sample (*p* ≤ 0.01) were carried forward in subsequent analyses.

### 2.11. Bioinformatics Analysis

We analyzed comprehensive TCGA cancer genome expression data by using UALCAN platform (http://ualcan.path.uab.edu/index.html). UALCAN is a publicly online website which can deeply analyses gene expression, promoter methylation, and correlation across defined clinicopathological features [28]. The mRNA expression level between normal tissue and cancer were analyzed from the Gene Expression Omnibus (GEO) data sets GSE32894 by using ShinyGEO online tool (https://gdancik.github.io/shinyGEO/). ShinyGEO is a web-based platform implemented using R package for building interactive web applications to analyze the difference of gene expression and survival outcome [29].

### 2.12. Statistical Analysis

IHC staining of tumor specimens was performed using anti-GAL1 antibody. The UTUC cell line (BFTC-909) was used for in vitro study of tumor invasiveness and migration. Kaplan–Meier analyses and Cox proportional regression models were used for univariate and multivariate survival analyses.

## 3. Results

### 3.1. LGALS1 mRNA Expression Increased Significantly in Advanced UC

**Figure 1:**
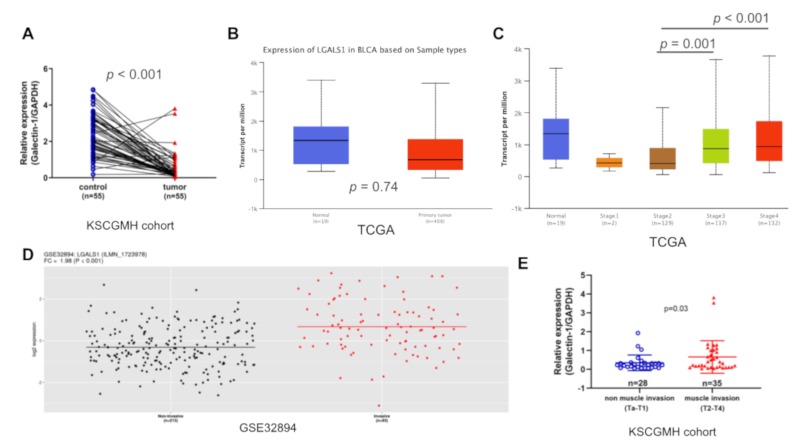
Galectin-1 (GAL1) expression was highly correlated with invasiveness of bladder cancer: (**A**) q-PCR analysis of GAL1 in paired normal bladder and cancer tissues in the Kaohsiung Chang Gung Memorial Hospital (KSCGMH) cohort. (**B**) Analysis of *LGALS1* expression in normal bladder tissues and cancer tissues by using the TCGA bladder cancer cohort (BLCA). (**C**) *LGALS1* expression in all stage of the BLCA cohort. (**D**) Analysis of LGALS1 expression in non-muscle invasion (Ta and T1) and muscle invasion (T2, T3, and T4) tumors by using GSE32894 data sets. (**E**) q-PCR analysis of GAL1 expression stratified by tumor invasiveness in the KSCGMH cohort.

To determine the extent of GAL1 expression in UC and normal tissues, we first examined the *LGALS1* mRNA levels in human bladder cancer tissues from the TCGA cohort and GSE32894 data set and our in-house q-PCR analysis of 55 paired normal tissue and cancer specimens (KSCGMH cohort). We found that the levels of *LGALS1* transcripts were significantly lower in tumor tissues than in the normal urothelium in KSCGMH cohort (*p* < 0.001; Figure 1A); however, this trend was not observed in the TCGA cohort (*p* = 0.74; Figure 1B); the difference in trend was possibly attributable to the small number of normal tissue samples (n = 19) in the TCGA cohort. Furthermore, high levels of *LGALS1* mRNA were expressed at the advanced stage of bladder cancer (stages 3 and 4) and not at the early stage (stages 1 and 2; Figure 1C) in the TCGA cohort. We further examined *LGALS1* expression in bladder cancers of different levels of invasiveness from the GSE32894 data set; *LGALS1* mRNA levels increased significantly in muscle invasion bladder tumors (*p* < 0.001; Figure 1D). Similarly, q-PCR analysis of all stages of bladder cancer revealed that GAL1 expression was higher in muscle invasion tumors than in non-muscle invasion tumors (*p* = 0.03; Figure 1E). Overall, GAL1 expression levels are strongly associated with bladder tumor stage and invasiveness.

### 3.2. High Expression of GAL1 is Associated with Poor Disease Recurrence and CSS

**Figure 2:**
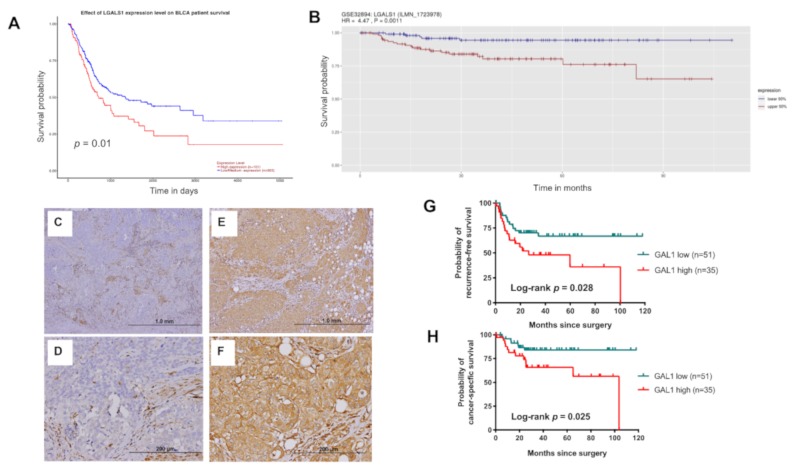
Evaluation of the association between GAL1 expression and oncological outcomes in urothelial carcinoma (UC): High expression of GAL1 reduced overall survival in patients with bladder cancer from the TCGA (**A**) and GSE32894 (**B**) cohorts. Representative immunohistochemical (IHC) images of low GAL1 (**C**,**D**) and high GAL1 (**E**,**F**) expression levels. Kaplan–Meier analysis of 86 patients with pT3 upper tract urothelial carcinoma (UTUC) for determining recurrence-free survival (RFS) (**G**) and cancer-specific survival (CSS) (**H**).

To understand the clinical effect of GAL1 expression in UC, we further investigated the differences in survival based on GAL1 expression levels in the TCGA BLCA and GSE32894 data sets. The overall survival of patients with tumors with high GAL1 expression was significantly poorer than that of patients with tumors with low GAL1 expression in the TCGA (*p* = 0.01) and GSE32894 cohorts (*p* = 0.0011; Figure 2A,B). To validate the findings and to explore the clinical importance of GAL1 expression in UTUC, we enrolled 86 patients with pT3 UTUC from the KSCGMH cohort for demographic and immunohistochemical (IHC) analysis. The median age was 71 years (interquartile range (IQR), 64–77). Among the 86 patients, 49 (57%) patients were female and 60% of patients had primary tumor located in the renal pelvis. Representative micrographs of GAL1 immunostaining are shown in Figure 2C–F. The study population was classified into two groups, namely GAL1-high (n = 35) and GAL1-low (n = 51) groups. The basic clinicopathological characteristics were comparable between the two groups (Table 1), and significant intergroup differences were not observed.

Within the median follow-up time of 40.4 months, 33 among 86 patients (38.4%) experienced disease recurrence and 26 (30.2%) patients died of disease. High GAL1 expression was significantly associated with a poor recurrence-free survival (RFS; *p* = 0.028; Figure 2G) and cancer-specific survival (CSS; *p* = 0.025; Figure 2H); hence, RFS and CSS were lower in the GAL-high group than in the GAL1-low group. After adjusting for all possible covariates by using Cox regression hazard models, high expression levels of GAL1 in UTUC tumors was an independent predictive factor for RFS (hazard ratio (HR) 2.43; 95% CI 1.17–5.05, *p* = 0.018) and CSS (HR 4.04; 95% CI 1.25–13.03, *p* = 0.019; Table 2). The other independent predictive factor for RFS and CSS was presence of lymphovascular invasion (LVI) (HR 2.41 for RFS; HR 3.56 for CSS).

### 3.3. Downregulation of GAL1 in UC Cell Lines

**Figure 3:**
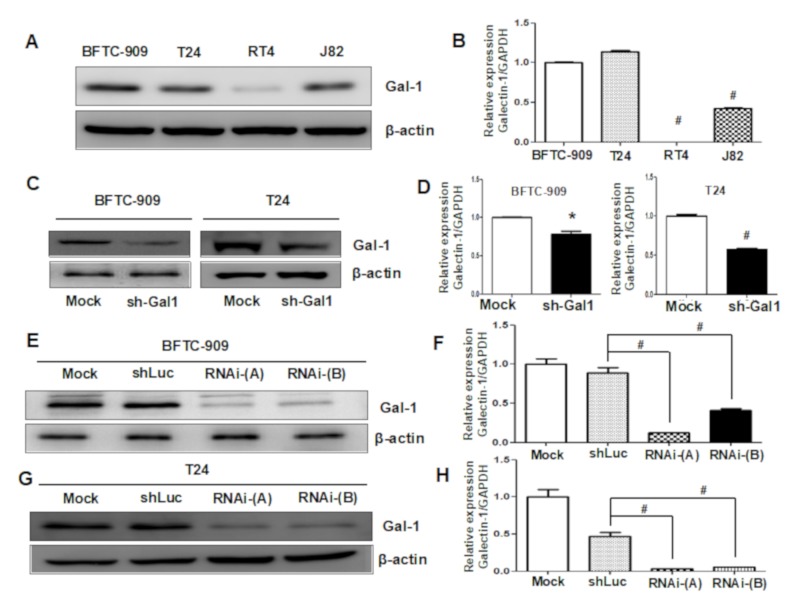
Evaluation of GAL1 expression in UC cell lines and verification of downregulation efficiency through Western blotting and q-PCR analyses: Data are presented as mean ± standard error of the mean from three independent experiments. (**A,B**) High expression of GAL1 in the BFTC-909 and T24 cell lines and low GAL1 expression in the J82 and T24 cell lines were observed. (**C,D**) The efficiency of GAL1 knockdown by using shRNA in the BFTC-909 and T24 cell lines was assessed through Western blotting and q-PCR analyses. (**E,F**) The efficiency of GAL1 knockdown by using lentiviruses was assessed through Western blotting and q-PCR analyses in the BFTC-909 cell line. (**G,H**) The efficiency of GAL1 knockdown by using lentiviruses was assessed through Western blotting and q-PCR analyses in the T24 cell line. * *p* < 0.05, # *p* < 0.001.

To determine the biological role of GAL1 in UC, we evaluated the expression levels of GAL1 in four UC cell lines (BFTC-909, T24, J82, and RT4) through Western blot and q-PCR analyses. As indicated in Figure 3A,B, the mRNA expression levels of GAL1 were highly upregulated in the BFTC-909, T24, and J82 cells and were positively correlated with high levels of GAL1 protein expression. The RT4 cell line exhibited low expression levels of GAL1 protein and mRNA. We selected the BFTC-909 and T24 cell lines, which exhibited the highest expression levels of GAL1, to construct GAL1 knockdown cell lines (sh-Gal1). As shown in Figure 3C,D, the levels of GAL1 protein and mRNA were significantly reduced in the sh-GAL1-T24 and sh-GAL1-BFTC-909 cells, indicating the efficiency of silencing *LGALS1* gene function. Furthermore, we established stable knockdown cell lines through the lentivirus transfection assay. As shown in Figure 3E–H, the amount of GAL1 protein and mRNA were significantly downregulated in the sh-GAL1-RNAi-A and sh-GAL1-RNAi-B BFTC-909 and T24 cells compared with shLuc and Mock. The knockdown efficiency of RNAi-A was higher than that of RNAi-B. 

### 3.4. GAL1 Expression Increased Tumor Invasiveness and Migration

**Figure 4:**
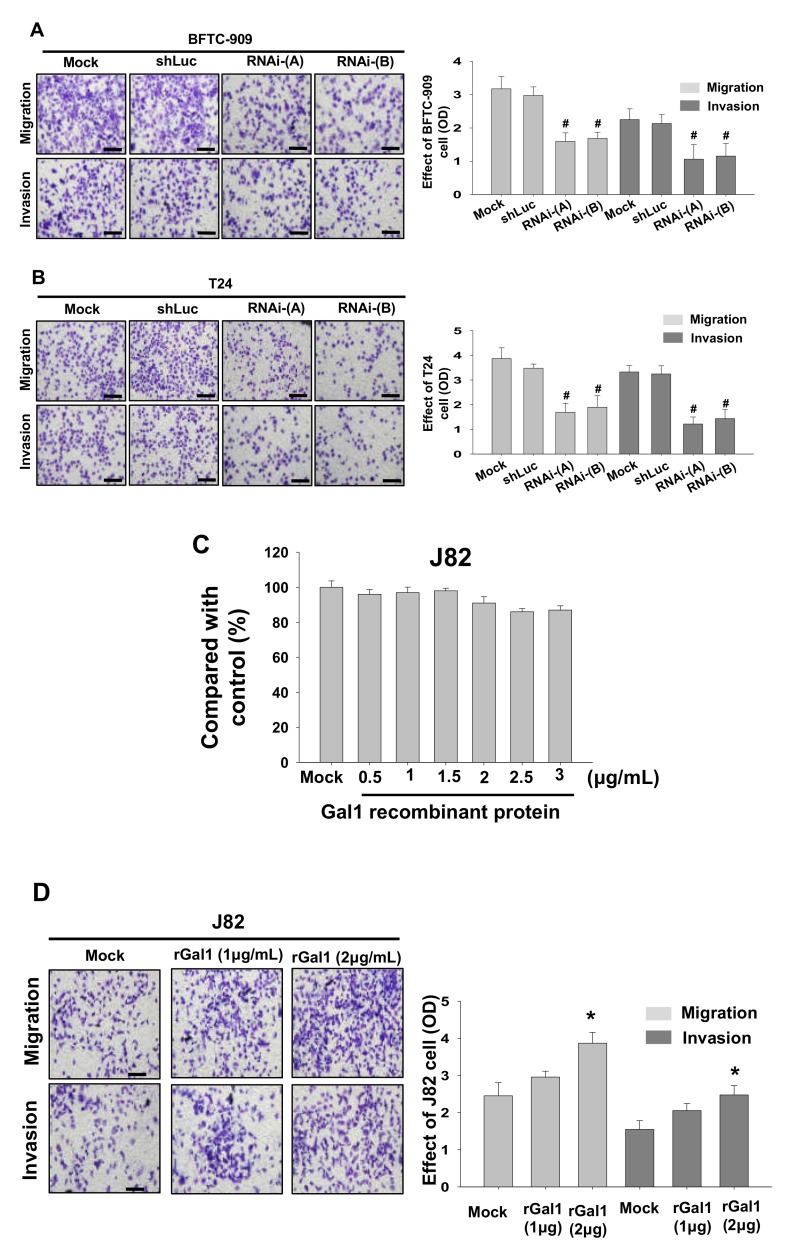
Downregulation of GAL1 affected cancerous behavior in the BFTC-909 and T24 cells and effects of different concentrations of GAL1 recombinant protein (0–3 μg/mL) on the J82 cells. GAL1 knockdown cells produced using lentiviruses exhibited a reduction in migration and invasiveness in the BFTC-909 (**A**) and T24 cells (**B**). (**C**) Viability of the J82 cells at different concentrations of GAL1 recombinant protein (0–3 μg/mL) after incubation for 24 h. (**D**) After 24 h of treatment with GAL1 recombinant protein (1–2 μg/mL), the percentages of migration of and invasion by the J82 cells significantly increased compared with the controls (Mock: cells treated with vehicle DMSO). Scale bar = 20 μm. * *p* < 0.05, # *p* < 0.001.

Next, we investigated tumor invasiveness of the GAL1 knockdown cells produced using lentivirus through transwell migration and invasion analysis. The results showed that lentivirus knockdown of GAL1 by using either RNAi-A or RNAi-B significantly reduced migration and invasion in the BFTC-909 and T24 cells (Figure 4A,B). Subsequently, we added GAL1 recombinant protein to the J82 cells for 24 h to verify the difference in migration and invasion assessed using the transwell method. The results showed the GAL1 recombinant protein (2.5–3 μg/mL) exhibited cytotoxicity (Figure 4C). Therefore, we selected 1 or 2 µg/mL as the concentration of GAL1 recombinant protein for subsequent analysis. The results showed that, after treatment with GAL1, recombinant protein, migration, and invasion increased significantly in the J82 cells (Figure 4D).

### 3.5. GAL1-Mediated Epithelial–Mesenchymal Transition in UC

**Figure 5:**
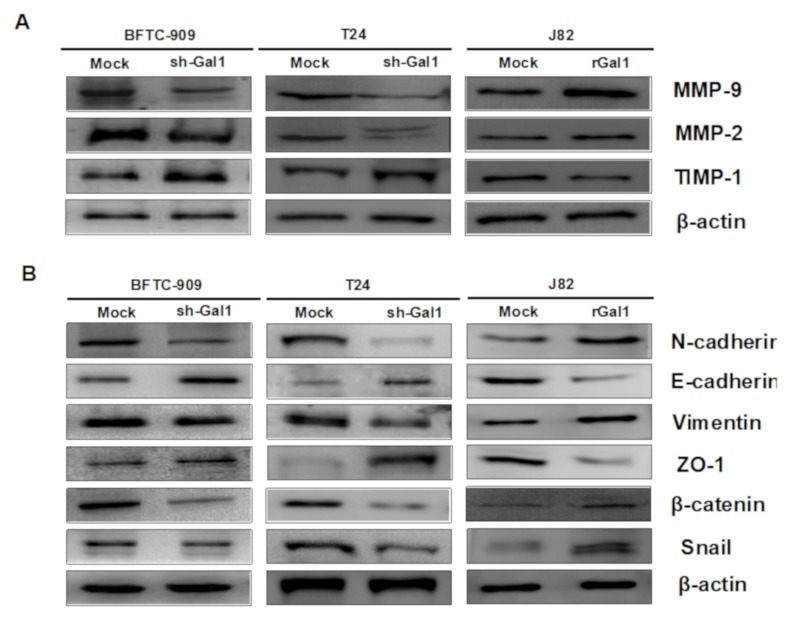
Effect of GAL1 on MMPs and EMT pathway proteins assessed through Western blotting analysis. Mock: cells treated with DMSO vehicle only. β-Actin was used as the protein loading control. (**A**) Total cell lysates of the BFTC-909, T24, and J82 cells treated with shRNA or GAL1 recombinant protein (1 μg/mL) were analyzed in terms of expression levels of MMP-2, MMP-9, and TIMP-1 by Western blotting. (**B**) Total cell lysates of the BFTC-909, T24, and J82 cells treated with shRNA or GAL1 recombinant proteins (1μg/mL) were analyzed in terms of expression levels of EMT pathway through Western blotting analysis.

MMP-2 and MMP-9 are well-known extracellular matrix (ECM)-degrading enzymes that have been reported to play crucial roles in cancer cell metastasis and invasion. We used Western blot analysis to investigate the effect of GAL1 knockdown or overexpression on associated protein levels of migration and invasion. The results showed that knockdown of GAL1 expression in the BFTC-909 and T24 cells significantly reduced the protein levels of MMP-2 and MMP-9 and increased TIMP-1 protein expression (Figure 5A), whereas increasing GAL1 expression in J82 cells by adding recombinant GAL1 protein reversed the phenomenon. EMT is one of the crucial mechanisms by which tumor cells detach from their primary site and invade surrounding tissues as well as the vascular system. We selected six representative molecules and assessed their expression levels in the BFTC-909 and T24 cells after GAL1 knockdown or overexpression. The results showed downregulation of N-cadherin, vimentin, β-catenin, and snail and upregulation of E-cadherin and ZO-1 after knockdown GAL1 in the BFTC-909 and T24 cells. The results of the J82 cells were contrary to the aforementioned results after the addition of GAL1 recombinant protein (Figure 5B).

### 3.6. Gene Expression Variations Among Samples

**Figure 6:**
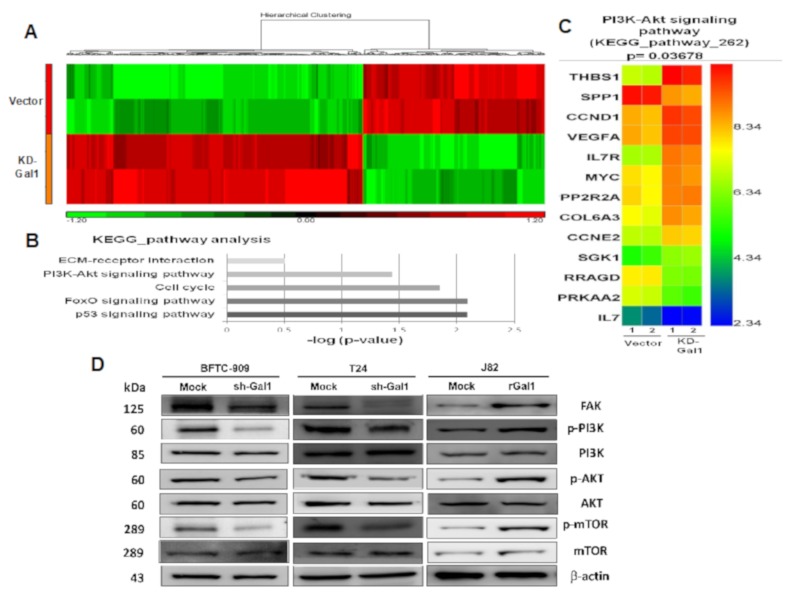
GAL1-mediated PI3K/AKT/mTOR pathway alteration assessed through microarray and Western blotting analyses: (**A**) A heatmap revealed upregulation and downregulation of genes with at least 2-fold difference between the GAL1-vector and GAL1-KD cells. (**B**) Top five gene enrichment pathways in KEGG pathway analysis. (**C**) Heatmap of the 13 altered genes in the PI3K/AKT signaling pathway in the BFTC-909 cells (GAL-vector and GAL1-KD). (**D**) Western blotting of GAL1 knockdown or overexpression in the FAK/PI3K/AKT/mTOR signaling pathways in the BFTC-909, T24, and J82 cells. Mock: Cells were treated with DMSO vehicle only. β-Actin was used as the protein-loading control.

To determine GAL1-associated gene alteration and downstream signal transduction pathways, we performed a high-throughput analysis by using Clariom D microarray to analyze two replicate samples (GAL1 vector and GAL1-KD). GAL1-mediated upregulation and downregulation of genes was depicted in a heatmap (Figure 6A), and all differentially expressed genes between the samples GAL1 vector and GAL-KD with at least fold change ≥2 and a nominal significance level of 0.01 are shown in Supplementary File S1 (Table S1). Subsequent KEGG pathway analysis was used to identify the top five gene enrichment pathways including p53, FOXO, cell cycle, PI3K/AKT, and ECM receptor signaling pathways (Figure 6B). We focused on gene alteration in the PI3K/AKT pathway; the results showed that *THBS1, CCND1, VEGFA, IL7R, MYC, PP2R2A, COL6A3,* and *CCNE2* were upregulated in GAL1-KD whereas *SPP1, RRAGD, PRKAA2,* and *IL7* were downregulated (Figure 6C). 

### 3.7. Downregulation of GAL1 Suppressed FAK and Phosphorylated PI3K-AKT-mTOR 

We further investigated the effects of GAL1 on the FAK/PI3K/AKT/mTOR pathways in vivo. The results showed that, in the GAL1 knockdown BFTC-909 and T24 cells, the phosphorylation of FAK, PI3K, AKT, and mTOR decreased. Moreover, in the J82 cells with GAL1 overexpression, the phosphorylation of FAK, PI3K, AKT, and mTOR increased. The protein expression of PI3K, AKT, and mTOR did not change after GAL1 knockdown or overexpression (Figure 6D).

## 4. Discussion

For treating patients diagnosed with pathological stage T3 UTUC, a consensus is not currently available for guiding clinicians to provide adjuvant therapy to prevent disease recurrence. Although several clinicopathological factors, such as LVI and tumor growth pattern, significantly affect prediction of disease recurrence, more precise and reliable biological markers are required to decipher mechanisms underlying UTUC progression and to guide medical practitioners. In the present study, GAL1 protein expression was highly associated with RFS and CSS in patients with UTUC. To our best knowledge, this is the first report to evaluate the prognostic value of GAL1 expression in patients with UTUC. We also demonstrated that downregulated GAL1 reduced tumor migration and invasion whereas addition of recombinant GAL1 protein resulted in increased malignant behavior in the J82 cells. Furthermore, we found that GAL1 mediated an increase in EMT, increased in MMP2/MMP9 activity, and altered the FAK/PI3K/AKT/mTOR pathway.

GAL1 is a homodimeric, β-galactoside-binding protein composed of 14.5 kDa subunits. GAL1 is abundantly expressed in various types of malignant tumors, including those in colorectal cancer [16], breast cancer [17], lung cancer [18], cancer of the uterine cervix [19], Hodgkin lymphoma [20], melanoma [30], ovarian cancer [31], and glioblastoma multiforme [32]. Regarding the clinical role of GAL1 expression in bladder cancer, Wu et al. demonstrated that overexpression of GAL1 in bladder tumors was significantly associated with higher pathological T grade and nodal stage as well as an increased risk of disease recurrence [25]. In the present study, we observed that 40% of pT3 UTUC tumors exhibited high expression of GAL1 (cutoff level was 10%). The clinicopathological features of UTUC with high or low GAL1 expression were not significantly different from each other. We further evaluated the prognostic role of GAL1 expression in UTUC patients through Kaplan–Meier analysis and by using Cox regression model. We found that increased expression of GAL1 protein by the IHC method was significantly associated with relapse or recurrence survival and CSS in both univariate and multivariate analyses, thus suggesting that GAL1 was an independent prognostic factor in determining oncological outcomes. Given that we selected patients with UTUC at the same pathological stage (pT3), the effect of the confounding factor of T stage was diminished, which reinforced the strength of the prognostic role of GAL1 in patients with pT3 stage UTUC. 

In humans, GAL1 is involved in many biological processes including cell adhesion, cell proliferation, invasion, migration, tumor angiogenesis, and immune escape [12,13,14,15]. Shen et al. elucidated the underlying molecular pathways of GAL1-mediated tumorigenesis in bladder cancer, mainly through the RAS-Rac1-MEKK4-JNK-AP1 pathway [26]. By using a comprehensive proteomic approach to investigate GAL1-regulated proteins, Li et al. showed that deregulated proteins are involved in several biological pathways, including lipid, amino acid, and energy metabolism; cell proliferation and apoptosis; cytoskeleton functions; cell–cell interaction; metastasis; and protein degradation [33]. Not only were several key proteins, such as fatty acid-binding protein 4 (FABP4), glutamine synthetase, toll interacting protein, and alcohol dehydrogenase NADP+ (AKR1A1), validated functionally by using immunoblotting methods but also their prognostic values were confirmed in a cohort study [33]. In the present study, we confirmed the results of the study by Shen et al., and our results showed that GAL1 expression was associated with tumor invasiveness and migration ability in UTUC (BFTC-909) and UCB (T24). We could successfully knockdown GAL1 expression in the BFTC-909 and T24 cell lines by using the shRNA and lentivirus methods, resulting in significant inhibition of tumor invasion and migration. By contrast, by adding recombinant GAL1 protein to cells with relatively low GAL1 expression (J82), tumor invasiveness and migration ability increased significantly, which indicated that GAL1 is a crucial factor for tumor aggressiveness and malignant behavior. 

The main intracellular pathway for GAL1 is through protein–protein interaction with H-RAS and activation of downstream MEK/ERK signaling [34]. GAL1 binds to Gemin4, which mediates the biogenesis of microRNA ribonucleoprotein and regulates pre-RNA splicing modulation [35]. However, the underlying signaling transduction pathway of GAL1-mediated UTUC carcinogenesis remains unknown. Through gene microarray analysis of parental and knockdown GAL1 cells, multiple crucial signaling pathways are involved in alteration of GAL1 expression, including the p53, FOXO, cell cycle, and PI3K/AKT pathways. We further validated microarray results through immunoblotting analysis, which showed that GAL1 regulated downstream FAK/PI3K/AKT/mTOR protein expression. In the previous study, Zhang et al. also showed that GAL1 induced hepatocellular carcinoma metastasis and resistance to sorafenib by upregulation of ανβ3-integrin and activated the PI3K/AKT pathway [36]. Our findings demonstrated comparable and consistent results, which indicated that GAL1-mediated tumor invasion and metastasis occurs through the FAK/PI3K/AKT/mTOR pathway.

## 5. Conclusions

**Figure 7:**
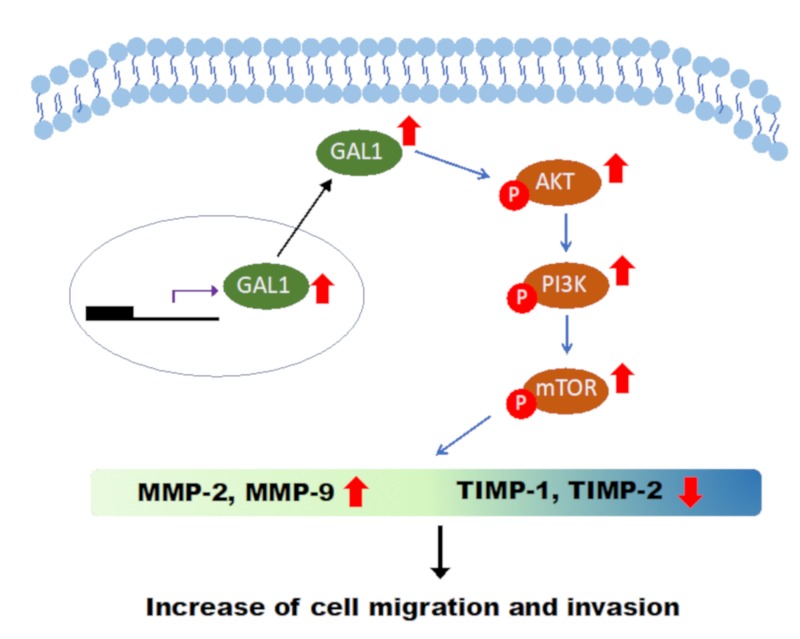
Representative Figure of GAL1-induced cancerous behavior in UTUC cells: Based on the results of our study, the cancerous behavior caused by GAL1 is mediated by the PI3K/AKT/mTOR pathway.

In brief, our study implied that the GAL1 protein is highly associated with oncological outcomes of UTUC by promoting tumor invasion, metastasis, and epithelial–mesenchymal transition, possibly through the FAK/PI3K/AKT/mTOR pathway (Figure 7).

## Figures and Tables

**Table 1:** Clinicopathological features of 86 patients with pT3 upper tract UC stratified by GAL1 expression.

	GAL1-Low	GAL1-High	*p*-Value
	(N = 51, %)	(N = 35, %)	
Age (years)	70.5 ± 10.8	68.0 ± 9.8	0.27
Gender			
Female	31 (60.8)	18 (51.4)	0.39
Male	20 (39.2)	17 (48.6)	
Smoking			
Yes	8 (15.7)	4 (11.4)	0.75
No	43 (84.3)	31 (88.6)	
Primary site			
Renal pelvis	26 (51.0)	26 (74.3)	0.09
Ureter	19 (37.3)	7 (20.1)	
Both	6 (11.8)	2 (5.7)	
Grade			
Low	0 (0)	1 (2.9)	0.41
High	51 (100)	34 (97.1)	
Histopathologic variant			
Presence	20 (39.2)	14 (40)	0.94
Absence	31 (60.8)	21 (60)	
CIS			
Presence	17 (33.3)	13 (37.1)	0.72
Absence	34 (66.7)	22 (62.9)	
LVI			
Presence	20 (39.2)	14 (40)	0.94
Absence	31 (60.8)	21 (60)	
Tumor necrosis			
Presence	18 (35.3)	16 (45.7)	0.33
Absence	33 (64.7)	19 (54.3)	
Multicentricity			
Yes	12 (23.5)	5 (14.3)	0.29
No	39 (76.5)	30 (85.7)	
Papillary feature			
Presence	31 (60.8)	15 (42.9)	0.10
Absence	20 (39.2)	20 (57.1)	

Abbreviations: CIS, carcinoma in situ; LVI, lymphovascular invasion.

**Table 2:** Univariate and multivariate analyses of RFS and CSS.

Characteristics	RFS			CSS		
	Univariate	Multivariate		Univariate	Multivariate	
	*p*-Value	HR (95% CI)	*p*-Value	*p*-Value	HR (95% CI)	*p*-Value
Age						
≥65 vs. <65	0.99			0.67		
Gender						
Male vs. female	0.17			0.41		
Smoking history						
Yes vs. no	0.16			0.08		
Primary site						
Kidney vs. ureter	0.24	2.03 (0.96–4.28)	0.06	0.23		
Histological variant						
Presence vs. absence	0.74			0.26	3.65 (1.20–11.12)	0.023
LVI						
Presence vs. absence	0.043	2.41 (1.15–5.05)	0.02	0.18	3.56 (1.06–11.88)	0.039
Tumor necrosis						
Presence vs. absence	0.25			0.35		
CIS						
Presence vs. absence	0.97			0.96		
Papillary feature						
Presence vs. absence	0.039			0.62		
Multicentricity						
Yes vs. no	0.74			0.82		
Galectin-1						
High vs. low	0.028	2.43 (1.17–5.05)	0.018	0.025	4.04 (1.25–13.03)	0.019

Abbreviations: CI, confidence interval; CIS, carcinoma in situ; HR, hazard ratio; CSS, cancer-specific survival; LVI, lymphovascular invasion; RFS, recurrence-free survival.

## Supplementary Materials

The following are available online at https://www.mdpi.com/2073-4409/9/4/806/s1. Supplementary File 1: Table S1. All differentially expressed genes (DEGs) between GAL1-vector and GAL1-KD UC cells.

## Abbreviations

CIS:	carcinoma in situ;
CSS:	cancer-specific survival;
ECM:	extracellular matrix;
EMT:	epithelial-mesenchymal transition;
FAK:	focal adhesion kinase;
GAL1:	galectin-1;
HR:	hazard ratio;
IHC:	immunohistochemical;
IQR:	interquartile range;
LVI:	lymphovascular invasion;
MMP:	matrix metalloproteinase;
mTOR:	mammalian target of rapamycin;
PI3K:	phosphoinositide 3-kinase;
q-PCR:	quantitative polymerase chain reaction;
RFS:	recurrence-free survival;
TCGA:	The Cancer Genome Atlas;
TIMP:	tissue inhibitors of metalloproteinases;
UC:	urothelial carcinoma;
UCB:	urothelial carcinoma of the bladder;
UTUC:	upper tract urothelial carcinoma.
