# Supplementary material for: Galectin-1 Overexpression Activates the FAK/PI3K/AKT/mTOR Pathway and Is Correlated with Upper Urinary Urothelial Carcinoma Progression and Survival
Source: Cells. 2020 Mar 26;9(4):806. doi: 10.3390/cells9040806 (PMC7226470; doi:10.3390/cells9040806)
Supplement: Supplementary file 1 [file cells-09-00806-s001.pdf]

cells

MDPI

Supplementary Table 1. All differentially expressed genes from microarray screening analysis.

Gene Symbol	Gene Name	FC[a] (Gal-KD versus Vector)	*p*-Value
	**Up-regulated genes**		
HAPLN1	hyaluronan and proteoglycan link protein 1	10.49	0.0027085
THBS1	thrombospondin 1	9.20	0.0022192
ODC1	ornithine decarboxylase 1	6.38	0.0055776
TGM2	transglutaminase 2	4.76	0.0015627
IL7R	interleukin 7 receptor	4.75	0.0017245
SERINC2	serine incorporator 2	4.51	0.0014919
ITM2C	integral membrane protein 2C	4.32	0.0044644
SERPINB7	serpin peptidase inhibitor, clade B, member 7	4.18	0.0081136
TNFRSF10D	tumor necrosis factor receptor superfamily, member 10d	4.01	0.0085561
TAGLN	transgelin	3.90	0.0099963
LRRN4	leucine rich repeat neuronal 4	3.82	0.0046513
TGFB2	transforming growth factor beta 2	3.51	0.0035017
CPA4	carboxypeptidase A4	3.43	0.0008452
EPB41L3	erythrocyte membrane protein band 4.1-like 3	3.34	0.0025309
NRG1	neuregulin 1	3.28	0.0079724
F3	coagulation factor III (thromboplastin, tissue factor)	3.27	0.0038968
POLR3G	polymerase III polypeptide G	3.26	0.0070675
SEMA7A	semaphorin 7A	3.20	0.0087335
NT5E	5-nucleotidase	3.17	0.0036353
CAMK2N1	calmodulin-dependent protein kinase II inhibitor 1	3.07	0.0090141
TIMP3	TIMP metallopeptidase inhibitor 3	3.03	0.0047953
SERPINE1	serpin peptidase inhibitor, clade E	2.97	0.0053652
MALL	mal, T-cell differentiation protein-like	2.88	0.0078205
DDAH1	dimethylarginine dimethylaminohydrolase 1	2.86	0.0002895
WDR3	WD repeat domain 3	2.85	0.0058842
WNT5A	Wnt Family Member 5A	2.81	0.0043796
GPR1	G protein-coupled receptor 1	2.81	0.0021313
HBEGF	heparin-binding EGF-like growth factor	2.80	0.0032597
EDIL3	EGF-like repeats and discoidin I-like domains 3	2.77	0.0064593
ICK	intestinal cell (MAK-like) kinase	2.74	0.0087263
NOL10	nucleolar protein 10	2.73	0.0066961
F2RL2	coagulation factor II (thrombin) receptor-like 2	2.71	0.0026477
MEST	mesoderm specific transcript	2.68	0.0047733
PNP	purine nucleoside phosphorylase	2.64	0.0006370
BCS1L	BC1 (Ubiquinol-Cytochrome C Reductase) Synthesis-Like	2.63	0.0045822
TIPIN	TIMELESS interacting protein	2.61	0.0018685
STC2	stanniocalcin 2	2.61	0.0020449
MYCBP	MYC binding protein	2.51	0.0085835
IL1RAP	interleukin 1 receptor accessory protein	2.50	0.0021069
ASNS	asparagine synthetase	2.46	0.0005380
AGPAT5	1-acylglycerol-3-phosphate O-acyltransferase 5	2.45	0.0077290
TES	testin LIM domain protein	2.44	0.0080561
ZNF92	zinc finger protein 92	2.42	0.0026145
RPF2	ribosome production factor 2 homolog	2.42	0.0060742
UBE2G2	ubiquitin conjugating enzyme E2G 2	2.42	0.0044759
CMSS1	cms1 ribosomal small subunit homolog	2.41	0.0013667
SLC7A5	Solute Carrier Family 7 Member 5	2.37	0.0070138

PDCD11	programmed cell death 11	2.36	0.0022351
EIF1AX	eukaryotic translation initiation factor 1A, X-linked	2.33	0.0035100
GTPBP10	GTP-binding protein 10	2.30	0.0026921
DKC1	dyskeratosis congenita 1	2.28	0.0074376
CCNE2	cyclin E2	2.28	0.0031590
KMT5B	lysine (K)-specific methyltransferase 5B	2.28	0.0099480
BCCIP	BRCA2 and CDKN1A interacting protein	2.27	0.0084604
LTBP1	latent transforming growth factor beta binding protein 1	2.26	0.0062012
EIF2S1	eukaryotic translation initiation factor 2, subunit 1	2.24	0.0062113
UBXN8	UBX domain protein 8	2.24	0.0000869
TAF9B	TAF9B RNA polymerase II	2.23	0.0040446
CMTM7	Chemokine-Like Factor Superfamily Member 7	2.23	0.0093194
NUP155	nucleoporin 155kDa	2.22	0.0044288
LTV1	LTV1 ribosome biogenesis factor	2.20	0.0013274
MARCH3	membrane associated ring finger 3	2.18	0.0062510
RPL7L1	ribosomal protein L7-like 1	2.18	0.0037954
ASUN	asunder spermatogenesis regulator	2.17	0.0077355
NUP43	nucleoporin 43kDa	2.16	0.0015478
WDR36	WD repeat domain 36	2.16	0.0076450
PPA1	pyrophosphatase 1	2.14	0.0045189
ACTBL2	actin, beta-like 2	2.14	0.0056570
FBXO5	F-box protein 5	2.14	0.0011233
NOL9	nucleolar protein 9	2.12	0.0056674
CALD1	caldesmon 1	2.11	0.0000290
PRDX3	peroxiredoxin 3	2.10	0.0033899
PDCD2L	programmed cell death 2-like	2.09	0.0030242
GNPNAT1	glucosamine-phosphate N-acetyltransferase 1	2.08	0.0068834
COBLL1	cordon-bleu WH2 repeat protein like 1	2.07	0.0064550
SLC19A1	solute carrier family 19 (folate transporter), member 1	2.07	0.0058154
MAMLD1	mastermind-like domain containing 1	2.06	0.0058814
NDUFAF5	NADH dehydrogenase complex I, assembly factor 5	2.06	0.0000048
NUP58	nucleoporin 58kDa	2.06	0.0029590
EIF3J	eukaryotic translation initiation factor 3, subunit J	2.05	0.0019420
C12orf45	chromosome 12 open reading frame 45	2.05	0.0072698
GPR89A	G protein-coupled receptor 89A	2.04	0.0099709
MTX3	metaxin 3	2.03	0.0090794
MMS22L	MMS22-like, DNA repair protein	2.02	0.0021267
CSTF2	cleavage stimulation factor, 3 pre-RNA, subunit 2	2.01	0.0043378

DNAJC2	DnaJ (Hsp40) homolog, subfamily C, member 2	2.01	0.0067042
**Down-regulated genes**			
TSHZ2	teashirt zinc finger homeobox 2	-2.00	0.0042495
ASAH1	N-acylsphingosine amidohydrolase (acid ceramidase) 1	-2.01	0.0007973
SCPEP1	serine carboxypeptidase 1	-2.03	0.0037456
GPC4	glypican 4	-2.05	0.0062917
TSPAN15	tetraspanin 15	-2.11	0.0051585
C10orf11	chromosome 10 open reading frame 11	-2.13	0.0043902
SOGA3	SOGA family member 3	-2.18	0.0033426
P2RX4	purinergic receptor P2X, ligand gated ion channel, 4	-2.29	0.0021858
SLC12A8	solute carrier family 12, member 8	-2.29	0.0058458
C3	complement component 3	-2.37	0.0056417
AKR1C3	aldo-keto reductase family 1, member C3	-2.47	0.0043377
ABCA1	ATP binding cassette subfamily A member 1	-2.58	0.0014356
SOD2	superoxide dismutase 2, mitochondrial	-2.59	0.0082673
NPR1	natriuretic peptide receptor 1	-2.66	0.0070771
ELOVL4	ELOVL fatty acid elongase 4	-2.77	0.0077961
AHR	aryl hydrocarbon receptor	-2.77	0.0041019
FADS2	fatty acid desaturase 2	-2.78	0.0074959
SPP1	secreted phosphoprotein 1	-2.82	0.0075980
SAMD12	sterile alpha motif domain containing 12	-2.98	0.0008378
RRAGD	Ras-related GTP binding D	-2.99	0.0009023
NMRK1	nicotinamide riboside kinase 1	-3.12	0.0032059
CYBRD1	cytochrome b reductase 1	-3.21	0.0083560
PROS1	protein S (alpha)	-3.25	0.0014517
**CHI3L2**	**chitinase 3-like 2**	**-3.44**	**0.00331867**
IFIH1	interferon induced, with helicase C domain 1	-3.53	0.0014771
IL18	interleukin 18	-3.83	0.00322251
SGPP2	sphingosine-1-phosphate phosphatase 2	-4.02	0.00059982
DRAM1	DNA-damage regulated autophagy modulator 1	-4.12	0.00144567
AICDA	activation-induced cytidine deaminase	-4.23	0.00448907
ANK3	ankyrin 3, node of Ranvier (ankyrin G)	-4.65	0.00223546
CPVL	carboxypeptidase, vitellogenic-like	-5.30	0.00142906
MAL2	mal, T-cell differentiation protein 2	-11.37	0.00274742

[a] Fold change(FC) between drug-resistant samples and -sensitive samples. Fold change with a positive value indicates a relatively higher concentration present in drug-resistant samples while a negative value means a relatively lower concentration compared to control samples.

According to the criteria of fold change ≥2 and nominal significance level of 0.01, 119 differentially expressed genes (DEGs) were identified in microarray between GAL1 vector and GAL1-KD samples, of which 86 were upregulated and 32 were downregulated.
